# Supplementary material for: Nanobubbles explain the large slip observed on lubricant-infused surfaces
Source: Nat Commun. 2022 Jan 17;13:351. doi: 10.1038/s41467-022-28016-1 (PMC8764024; doi:10.1038/s41467-022-28016-1)
Supplement: Supplementary file 1 — Supplementary Information [file 41467_2022_28016_MOESM1_ESM.pdf]

Supplementary Information:
Nanobubbles explain the large slip observed on lubricant-infused surfaces

Christopher Vega-Sánchez
School of Chemistry and University of Sydney Nano Institute, The University of Sydney, NSW 2006 Australia
University of Sydney Nano Institute, The University of Sydney, NSW 2006 Australia and
School of Electromechanical Engineering, Costa Rica Institute of Technology, Campus Cartago, 159-7050 Costa Rica

Sam Peppou-Chapman, Liwen Zhu, and Chiara Neto
School of Chemistry and University of Sydney Nano Institute,
The University of Sydney, NSW 2006 Australia and
University of Sydney Nano Institute, The University of Sydney, NSW 2006 Australia

MATERIALS AND METHODS

Microfluidic devices

Two custom-built microfluidic devices were used, both with five independent microchannels (Fig. S1). One device was made of PMMA (Fig. S1a) and one of aluminum (Fig. S1b), both with the same channel geometry. The width of the channel W, measured using a contact profilometer Tencor P11 in three different places, was found to be 2.502 $\pm$ 0.005 mm. Accurate channel height H measurements were made after each experiment as described later. Both devices were cleaned thoroughly prior to use. A thin layer of PDMS (1-2 μm, Sylgard 184) was used to seal the devices, as described in [1].

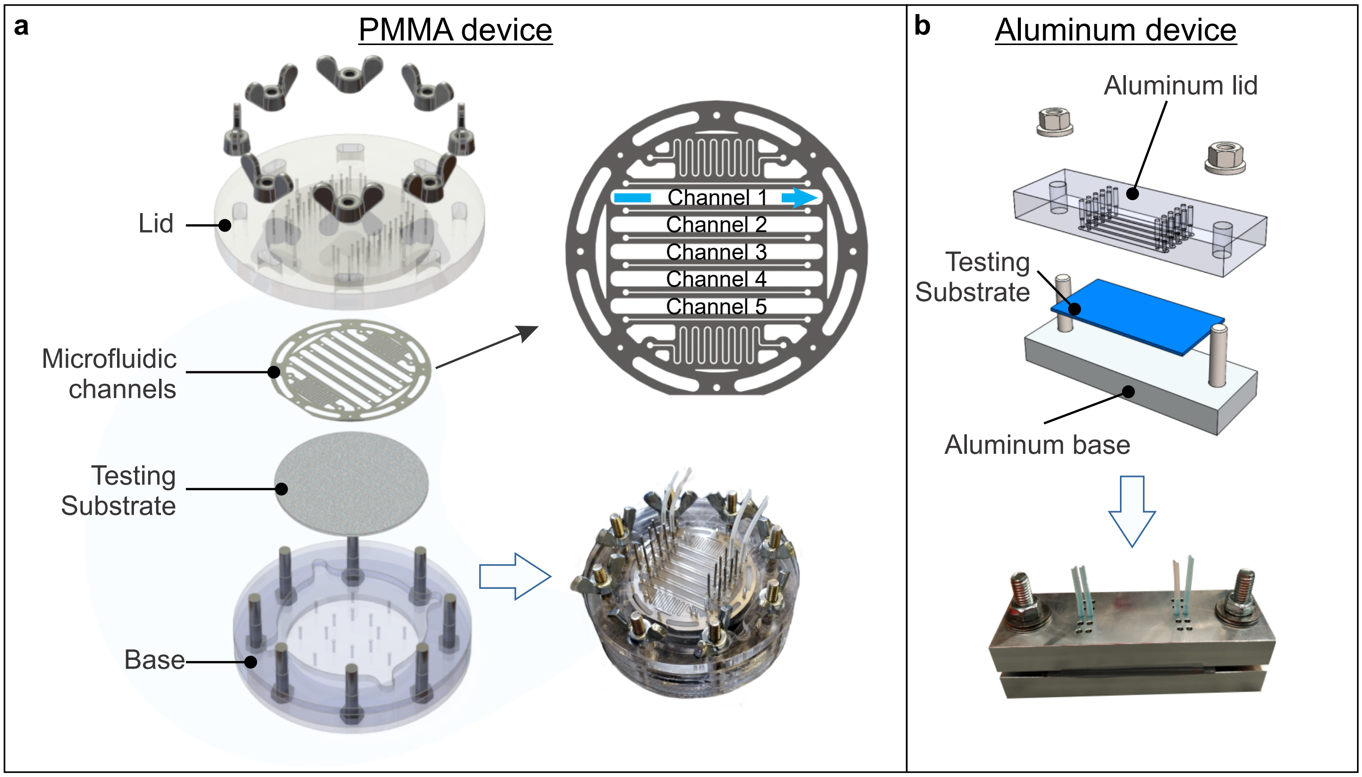

FIG. S1. Exploded view of the modular microfluidic devices made of (a) PMMA and (b) aluminum. Both devices contain five straight channels of same size and geometry. The last images are pictures of the real device.

For all the LIS, the lubricant used was either silicone oil (5 or 10 cSt, Sigma Aldrich) or hexadecane (Sigma Aldrich). The working fluid (external fluid) was either Milli-Q water or mixtures of glycerol and water to produce viscosity

ratios μ_w/μ_o of 0.1, 0.2, 5.5 and 9.7. The fluid properties are shown in Table S1. Table S2 shows the number of experiment repetitions conducted for each substrate.

TABLE S1. Physico-chemical properties of fluids used at 25 °C.

Fluid	Density[a] ρ (kg m^{-3})	Dynamic Viscosity[b] μ (mPa s)	Interfacial tension[c] working fluid/ silicone oil 10 cSt γ (mN s^{-1})	Interfacial tension[c] working fluid/ Hexadecane γ (mN s^{-1})
Silicone oil 10 cSt (as lubricant)	930	9.30	-	-
Silicone oil 5 cSt (as lubricant)	913	4.57	-	-
Hexadecane (as lubricant)	773	3.00	-	-
Water (Milli-Q)	997	0.89	42.1 ± 0.1	52.7 ± 0.3
55% Glycerol-water mixture	1153	9.30	29.4 ± 0.3	41.6 ± 0.7
70% Glycerol-water mixture	1192	27.9	29.1 ± 0.3	37.0[d]
78% Glycerol-water mixture	1209	51.00	29.2 ± 0.2	34.9 ± 0.3
83% Glycerol-water mixture	1220	90.10	30.6 ± 0.3	32.1 ± 0.4

[a] Measured using a density meter (DMA 35N, Anton Paar). Density accuracy: 0.001 g cm^{-3}. Temperature accuracy: 0.2 °C.
[b] Measured using a modular compact rheometer (MCR 302, Anton Paar) with a concentric cylinder CC27. Temperature accuracy: 0.01 °C.
[c] Measured through the pendant drop method on a tensiometer CAM200 (KVS Instruments). The error is the standard deviation of at least 12 samples.
[d] Measurement was not possible due to refractive index matching between the fluids. This value was obtained by a linear approximation from neighboring points.

TABLE S2. Number of experiment repeats conducted for each substrate and for each microfluidic device.

Testing Substrate	Lubricant	Working fluid	PMMA device	Al device
Si-wafer	-	water	10	5
OTS-Si wafer	-	water	10	0
OTS-Si wafer infused	Silicone oil (10 cSt)	water	7	0
PDMS-Si wafer	-	water	0	6
PTFE	-	water	5	4
Teflon Wrinkles	-	water	7	5
Teflon Wrinkles	Silicone oil (5 cSt)	water	5	10
Teflon Wrinkles	Silicone oil (10 cSt)	water	9	13
Teflon Wrinkles	Silicone oil (10 cSt)	55% Gly mix	5	5
Teflon Wrinkles	Silicone oil (10 cSt)	78% Gly mix	5	7
Teflon Wrinkles	Silicone oil (10 cSt)	83% Gly mix	5	10
Teflon Wrinkles	Hexadecane	water	5	8
Teflon Wrinkles	Hexadecane	55% Gly mix	0	9
Teflon Wrinkles	Hexadecane	70% Gly mix	0	8

Air content in water

Air content in water was changed by placing the water under air pressure for at least 30 min and up to 4 hours using a 50 mL-Falcon tube, as shown in Fig. S2. The volume of water used was approximately 20 mL at a time, to minimise gassing times. As shown in Fig. S2a-b, the tube was positioned horizontally in order to increase the water-air interface area and was shaken at intervals of approximately 10 min. In order to quantify the air content in water after the gassing or degassing procedure described above, we measure the amount of dissolved oxygen using an oxygen sensor (RCYACO, Model DO9100).

The sensor measures simultaneously oxygen content and temperature. The proper operation of the sensor was tested by measuring the oxygen content in air-saturated water and deoxygenated water, which were produced by placing 300 mL of Milli-Q water under a continuous flow of air and high-purity nitrogen, respectively, for 1 hour. According with [2] the latter is the most effective way to remove oxygen from water. The air and nitrogen flow rates were measured by counting the time required to displace the water in an upturned measuring cylinder full of water. The air and nitrogen pressures were adjusted to achieve an approximate flow rate of 200 mL s^{-1}. Oxygen

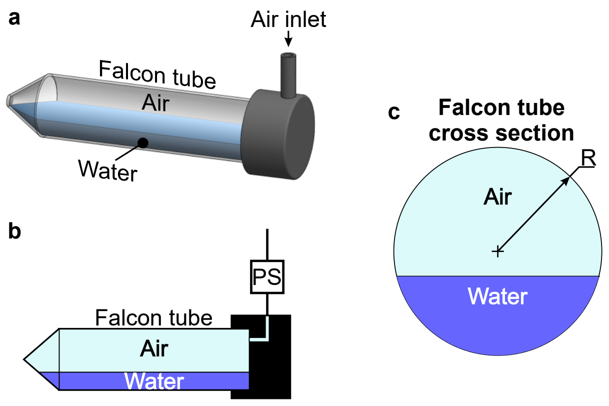

FIG. S2. Schematic of the setup used to change the gas content in water. (a) Schematic (made at scale) of Falcon tube containing 20 mL of water. (b) Longitudinal and (c) transverse cross sections. PS: Pressure sensor. R: inner radius of Falcon tube ≃ 12.5 mm.

TABLE S3. Dissolved oxygen concentration C_{O_2} in air-saturated water and deoxygenated water at 1 atm.

Measurement	Experimental C_{O_2} (mg/L)	Experimental saturation (%)	Experimental Temperature (°C)	Theoretical saturation C_{O_2} (mg/L)	Theoretical saturation (%)
			**Air-saturated water**		
1	10.0	99.7	15.0	10.1	99.2
2	9.8	98.1	14.7	10.1	96.6
3	9.8	97.6	14.5	10.2	96.1
4	10.0	98.7	14.6	10.2	98.3
**Average**	**9.9**	**98.5**	**14.7**	**10.1**	**97.5**
**SD**	**0.1**	**0.9**	**0.2**	**0.0**	**1.4**
			**Deoxygenated water**		
1	0.0	0.3	14.6	10.2	0.0
2	0.1	0.8	15.0	10.1	1.0
3	0.1	0.2	14.7	10.1	1.0
4	0.0	0.0	15.4	10.0	0.0
**Average**	**0.1**	**0.3**	**14.9**	**10.1**	**0.5**
**SD**	**0.1**	**0.3**	**0.4**	**0.1**	**0.6**

measurements were taken every 5 min, alternating between air-saturated water and deoxygenated water. Table S3 shows the measurements. The temperature measurement given by the sensor was found to be within 1 °C of that taken by a T-type thermocouple. The theoretical value of the oxygen concentration C_{O_2} given in mg L^{-1} at 1 atm and corrected by temperature was estimated using the following equation (a corrected version of Henry's law for temperature other than 25°C)): [3]

$$C^*_{O_2} = -139.34410 + \frac{1.575\,701 \times 10^5}{T} - \frac{6.642\,308 \times 10^7}{T^2} + \frac{1.243\,800 \times 10^{10}}{T^3} - \frac{8.621\,949 \times 10^{11}}{T^4} \quad (1)$$

where T is the temperature given in K. The measurements presented in Table S3 are in good agreement with the theoretical predictions and validate the correct operation of the sensor. For air-saturated water and deoxygenated water, the percentage of saturation was found to be $98.5 \pm 0.5\%$ and $0.3 \pm 0.3\%$, respectively.

The oxygen saturation measured in Milli-Q water as supplied by the dispenser was found to be $97.6 \pm 0.7\%$ (over six measurements). The oxygen content over time was measured for degassed, gassed and highly gassed water as presented in Table S4. The expected saturation oxygen concentration value, corrected by temperature and pressure, was estimated using the following equation: [3]

$$C_{O_2} = C^*_{O_2} P \left[\frac{(1 - P_{WV}/P)(1 - \theta P)}{(1 - P_{WV})(1 - \theta)} \right], \quad (2)$$

TABLE S4. Dissolved oxygen concentration in Milli-Q water exposed to different air pressures over time.

Absolute pressure (kPa)	Time (min)	Experimental C_{O_2} (mg/L)	Temperature (°C)	Theoretical saturation C_{O_2} (mg/L)	Saturation (%)
6 (Degassed water)	0	8.8	19.5	0.35	2540
	10	2.3	17.4	0.39	593
	20	1.4	16.5	0.41	345
	30	0.7	16.8	0.40	175
	70	0.9	14.9	0.44	206
203 (Gassed water)	0	8.8	19.5	18.6	47.0
	10	10.5	19.8	18.5	56.8
	20	13.4	19.6	18.6	72.2
	30	15.4	19.8	18.5	83.3
	70	16.6	19.8	18.5	89.8
304 (Highly gassed water)	0	8.8	19.5	28.0	31.7
	10	12.9	18.8	28.3	45.5
	20	20.2	18.9	28.3	71.5
	30	22.3	18.9	28.3	78.9
	70	23.7	18.7	28.4	83.5

where P_{WV} is the water vapor pressure in atm calculated as $\ln P_{WV} = 11.8571 - 3840.70/T - 216961/T^2$ and θ is the negative of the second pressure coefficient in the virial expansion for the real gas behavior of oxygen estimated as $\theta = 0.000975 - 1.426 \times 10^{-5}(T - 273.15) + 6.436 \times 10^{-8}(T - 273.15)^2$. Here T is the temperature given in K.

As observed in Table S4, after 30 min, the oxygen concentration in the degassed water was reduced from 8.8 to 0.7 mg L^{-1}, corresponding to a 92% reduction with respect the original gas concentration. In water exposed to an air pressure of 203 and 304 kPa, the oxygen concentration reached a saturation of 83% and 79%, respectively. Additionally, we found that if we repeated the experiment without opening the Falcon tube to make oxygen measurements every 10 min, the saturation values were higher after 30 min, corresponding to saturation percentages of 92% and 84% for pressures of 203 and 304 kPa, respectively.

Considering that in water at 20 °C the oxygen to nitrogen ratio is 38:62, the concentration of air in water according to the pressure applied is shown in Table S5. Given that the pressure was applied for a period between 30 min and up to 4 h, the values presented in the table correspond to average value between the experimental measurement at the 30 min-mark and the saturation value estimated by Eq. 2. The units in the table are in mg L^{-1}, however, given that the density of water at 20 °C is 998.2 kg m^3, the conversion produces the same values in mg kg^{-1}.

TABLE S5. Dissolved oxygen C_{O_2} and air C_{air} concentration in Milli-Q water at a given pressure and 20 °C.

	Absolute pressure (kPa)	Experimental C_{O_2} (mg/L)	Experimental C_{air} (mg/L)	Theoretical C_{air} saturation (mg/L)	Saturation (%)
Degassed water	6	0.5 ± 0.2	1.4 ± 0.5	0.9	154
Milli-Q water (as produced)	101	8.8 ± 0.1	23.0 ± 0.3	23.7	97
Gassed water	203	16.9 ± 1.5	44.1 ± 3.9	48.2	92
Highly gassed water	304	25.0 ± 2.7	65.2 ± 7.0	72.4	90

Channel height measurement

The channel height H has a strong influence on the correct estimation of $\Delta p_{no-slip}$, so after each experiment, each channel height was measured. An exact PDMS replica of each individual channel was molded without opening the device. The PDMS cured within each channel at room temperature for 72 hours. The replicas were then placed on a glass slide and the height of each was measured in three different locations using a contact profilometer (Fig. S3). Cross-sections were cut and imaged optically with a 50x objective. The average of the profilometry data was used as the channel height H, with a standard deviation for H was of 0.7 - 1 µm along the 25 mm length L.

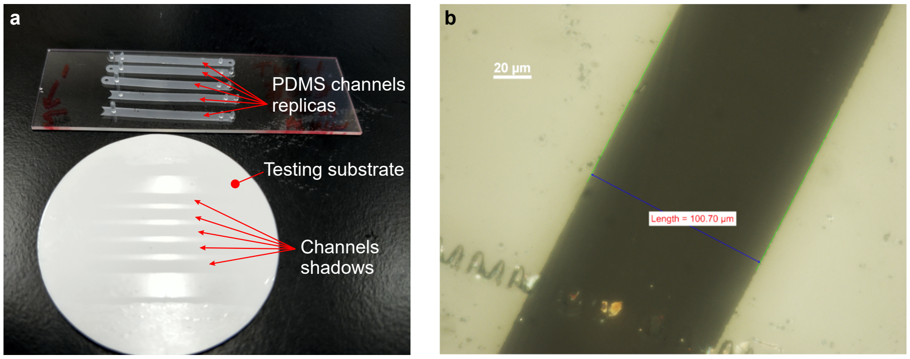

FIG. S3. (a) PDMS replica of the channels placed on a glass slide. On the testing substrate the channel shadows can be observed. (b) Typical optical micrograph of a replica cross-section imaged using a 50x objective.

Validation of experimental setup on smooth substrates

A hydrophilic plasma-cleaned silicon wafer was used to validate the experimental setup under no-slip boundary conditions. In Fig. S4a, the experimental pressure drop values for water flowing over silicon wafer are compared with the expected pressure drop derived from the standard friction factor equation for the no-slip condition (dashed line). The agreement between the experimental values and the theory is evidence of the correct operation of the setup.

Smooth hydrophobic (octadecyltrichlorosilane-coated, OTS) silicon wafers were tested under the same hydrodynamic conditions, see Fig. S4b,c. As stated in the text, the slip length on these surfaces is expected to be too small to be measured with our microfluidic setup [4].

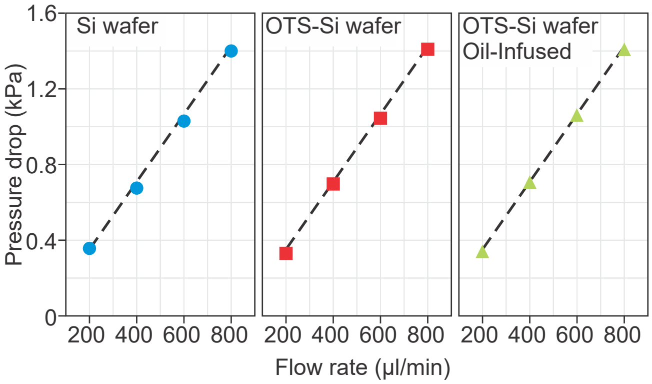

FIG. S4. Pressure drop measurements as a function of flow rate of water for hydrophilic silicon wafer (blue dots), hydrophobized (OTS-coated) silicon wafer (red squares) and 10-cSt-silicone oil-infused OTS-coated silicon wafer (green triangles). The dashed black line represents the no-slip pressure drop derived from the friction factor equation.

Characteristic length of wrinkled Teflon substrates

The Teflon wrinkles were fabricated by thermally annealing a Teflon film over a shrinkable substrate [5]. The resulting topography is random but uniform over large areas. AFM micrographs were analyzed using Matlab in order to estimate the characteristic length s between the peaks of the wrinkles. Cuts made h=-200 nm below the highest peaks reveal the presence of large pools of oil trapped between the wrinkles, with a average distance s between peaks of 13 $\pm$ 5 µm and an exposed area of only 0.3% (Fig. S5b). The characteristic distance s between peaks (pool size) has a bimodal distribution around 2.5 and 13 µm (inset in Fig. S5b).

When the infused oil depletes from the substrates, more peaks may become exposed. A second lower cut (Fig. S5c) reveals an average distance s between peaks reduces to 7 $\pm$ 2 µm and the exposed area increases to 8%, with a

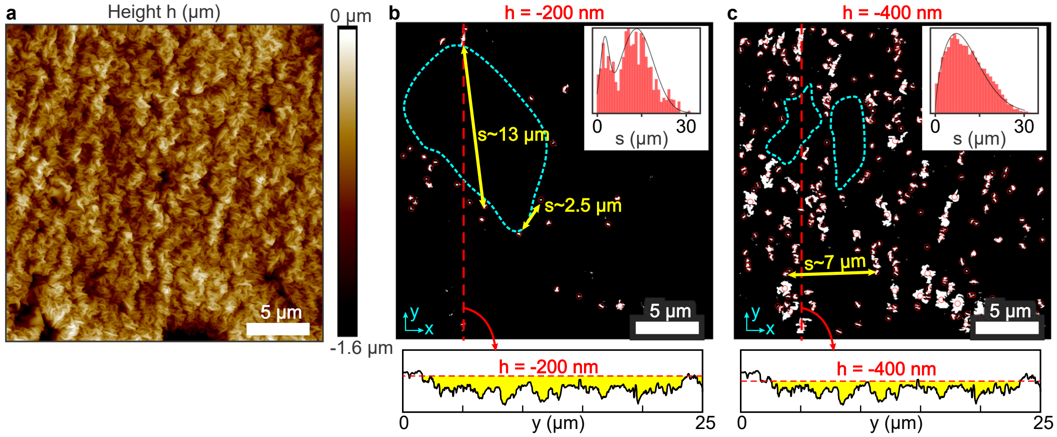

FIG. S5. Analysis of the topography of the Teflon wrinkles. (a) AFM micrograph. (b) Image thresholding was applied to (a), so in white are areas in a cut plane at 200 nm and (c) 400 nm below the highest peak in the wrinkles, as shown in the cross-sectional profiles below (b) and (c) with the dashed red line. The yellow arrows show the characteristic length s and the dotted cyan lines illustrate the lubricant pools for each case. Insets are histograms of the distribution of characteristic length s for each image.

transition from a bimodal distribution of the pool sizes (inset in Fig. S5b) to a monomodal distribution (inset in Fig. S5c).

ERROR IN THE MEASUREMENT OF DRAG REDUCTION

The accuracy with which the drag reduction factor (Eq. 3 in main manuscript) can be estimated depends on the accuracy of the experimental pressure drop measurement $\Delta p_{no-slip}$, and more critically on the channel height H and the temperature T (Eq. 2 in main manuscript). Each micrometer of deviation in the determination of H will introduce an error of 3% in the estimation of $\Delta p_{no-slip}$. On the other hand, each degree of deviation in the measurement of the fluid temperature will lead to an error of 2.3%. In this experiment, the uncertainty of each measurement was minimised and the propagation of all the errors will induce a overall error $\sigma_{p_{ns}}$ which can be estimated as follows:

$$\sigma_{p_{ns}}^2 = \left(\frac{\partial \Delta p_{ns}}{\partial W}\right)^2 \sigma_W^2 + \left(\frac{\partial \Delta p_{ns}}{\partial H}\right)^2 \sigma_H^2 + \left(\frac{\partial \Delta p_{ns}}{\partial L}\right)^2 \sigma_L^2 + \left(\frac{\partial \Delta p_{ns}}{\partial T}\right)^2 \sigma_T^2 + \left(\frac{\partial \Delta p_{ns}}{\partial Q}\right)^2 \sigma_Q^2 \tag{3}$$

TABLE S6. Uncertainty in the measurement of experimental variables.

Variable	Units	Expected value	Mean value	Standard deviation
Channel Width W[a]	mm	2.502 ± 0.005	2.49	0.01
Channel Height H[a]	μm	100.3 ± 0.1	100.4	0.5
Channel Length L[b]	mm	25.01 ± 0.01	24.99	0.01
Temperature T[c]	°C	0 ± 1	0.1	0.2
Flow rate Q[d]	$\mu L\,min^{-1}$	800 ± 5	801.4	1.2

[a] Sample size $n = 8$. Measurements were made with a contact profilometer Tencor P11.
[b] Sample size $n = 12$. Measurements were made with a digital caliper Mitutoyo series 500 with a resolution of 0.01 mm.
[c] A bath of water with ice was used as calibration reference temperature. A high accuracy thermocouple type T was used to measure the temperature every minute from time 0 to 5 min and then every 5 minutes. Sample size $n = 10$. The bath temperature was constantly checked using a mercury thermometer with a resolution of 0.1 °C.
[d] The sensor was calibrated using the mass method with a precision balance with a resolution of 0.0001 g. Sample size $n = 5$.

Table S6 shows the calibration of the instruments used for the estimation of each variable. Therefore, using Eq. 3, the error of our estimation of $\Delta p_{no-slip}$ ranges between 1.8 and 1.9% for flow rates between 800 $\mu L\,min^{-1}$ to 200 $\mu L\,min^{-1}$, respectively. Pressure sensors with a specified resolution of 20 Pa. The calibration of the sensors was verified by comparing the reading of the sensor with respect to a water column manometer with a resolution of 2.5 Pa

and in all cases the measurement was within range. Therefore, the error propagation for the D_R was estimated to be 2 and 5% for flow rates between 800 µL min^{-1} to 200 µL min^{-1}, respectively.

Using Eq. 3 and 4 in the main manuscript, D_R and b were computed for each individual experiment, i.e. specific flow rate, specific microfluidic channel and specific substrate. Then, these values were averaged to calculate the final effective slip lengths with standard deviation.

LUBRICANT STABILITY ON WRINKLED TEFLON SUBSTRATES UNDER FLOW

Pressure drop was measured over 24 hours on the infused surfaces and remained constant (Fig. S6). Small fluctuations are due to temperature variation during the experiment, and have standard deviation smaller than 2%. The average drag reduction is 12 ± 1, 22 ± 2, 27 ± 2% for viscosity ratio of 0.1, 5.5 and 9.7, respectively. These results demonstrate that the lubricant film is stable under the imposed flow rate and are consistent with the confocal micrographs (Fig. S7b).

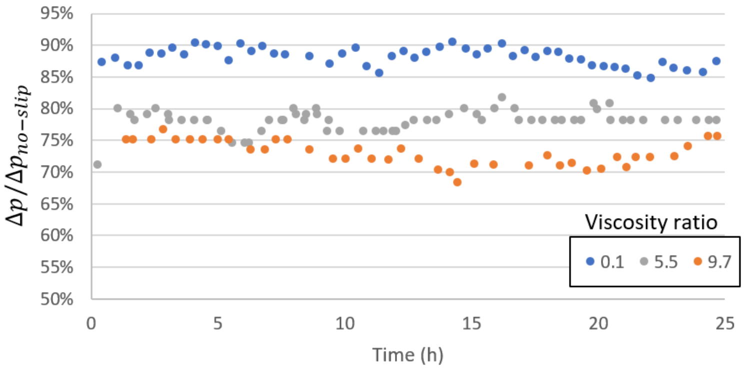

FIG. S6. Pressure drop measurements versus time for Teflon wrinkles infused with silicone oil (10 cSt) using water and water-glycerol mixtures ($c_{air} \sim 44 \pm 4$ mg kg^{-1}) to achieve the corresponding viscosity ratio.

The stability of the lubricant layer was studied under flow. After 30 minutes of flow at each flow rate (from 200 to 800 µL min^{-1}), the fluorescent signal shows that the thickness of the silicone oil layer diminishes stepwise with increasing flow rate (Fig. S7a). However, after an hour of flow at a fixed flow rate (800 µL min^{-1}), the intensity of the emission decreases only slightly, likely due to bleaching (Fig. S7b), indicating that the lubricant is stable and does not deplete over time.

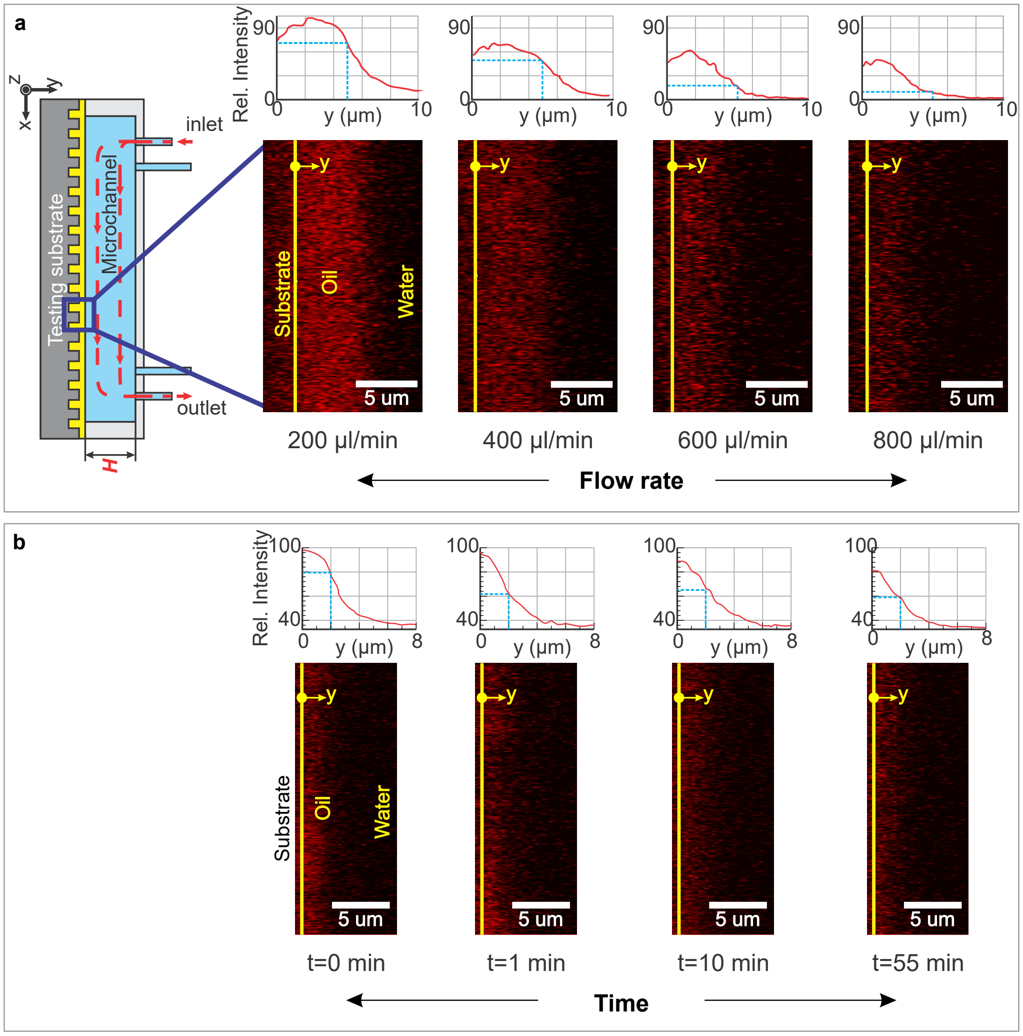

FIG. S7. (a) Confocal microscopy results showing the emission of the lubricant under different flow rates of water ($c_{air} \sim 23.0 \pm 0.3$ mg kg^{-1}). The insets show the relative fluorescence intensity vs distance from the surface. The blue dashed lines are a guide to highlight the intensity decay with increasing flow rate. (b) Emission of the lubricant over time at a fixed flow rate of 800 µL min^{-1}. The vertical scale of the insets has been modified to appreciate better the minimal change in the intensity of the emission.

Lubricant film mapping using meniscus force atomic force microscopy

It is estimated that the lubricant thickness under water is of the order of 1 µm, corresponding to a total volume of lubricant per channel of ∼ 100 nL. After exposure to a flow rate of 800 µL min^{-1}, the total lubricant loss per channel was found to be ∼ 60 nL. This small volume means that any depleted lubricant accumulated within the channel during the experiment would not alter the flow conditions.

Fig. S8 shows the topography of the wrinkles, a map of the oil distribution on the wrinkles after exposure to flow at each rate, and a histogram of lubricant thickness distribution at each stage. The regions in purple in the maps in the second column of the figure represent exposed areas (where the lubricant thickness is less than 10 nm). The histograms represent the frequency with which each lubricant thickness is present in the surface. The gradual decrease in thickness with increasing flow rate suggests that the lubricant depletion does not follow a linear trend with respect to the flow rate.

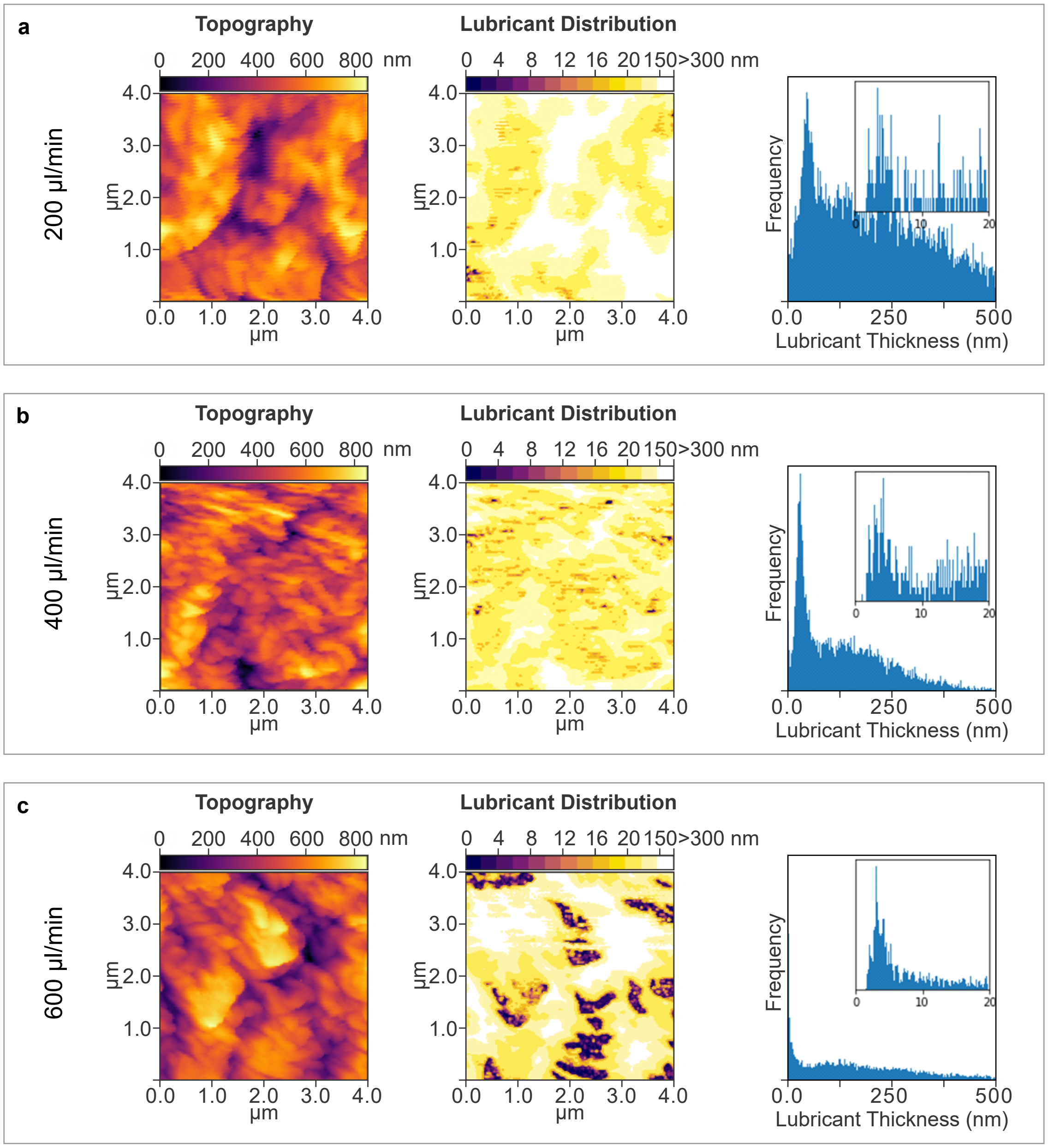

FIG. S8. Lubricant thickness mapping using meniscus force AFM measurements in air, after exposing the surface to flow of gassed water ($c_{air} \sim 44 \pm 4$ mg kg^{-1}) for 30 min at (a) 200, (b) 400 and (c) 600 µL min^{-1}. From left to right, each set of images shows the wrinkled surface topography, the lubricant film thickness and the histogram of the lubricant distribution. These maps were collected in air (not underwater) with the aim to quantify only the lubricant depletion.

BUBBLE NUCLEATION

Laser confocal scanning microscopy of bubble nucleation

Fig. S9 and Fig. 4 in the main text show that microscale bubbles nucleate readily on these substrates, they grow within a few seconds to several tens of µm and that the presence of the lubricant is not a barrier for this to occur. We found that: 1) nucleation can take place both under flow or in the absence of it and, 2) that the air content in the water affects the final bubble size. Under flow the microbubbles appear more quickly (normally within minutes) than when the substrate is simply immersed with no flow (normally half an hour or more). This could be an indication of air diffusion through the lubricant layer and a redistribution of lubricant under flow. Even in presence of flow, bubbles

remain on the substrate until they reach a significantly larger size to be dragged by the flow. If the flow stops once the bubble is formed, the bubble remains pinned on the substrate and the growth rate slows down.

Mapping of nanobubbles on oil-infused Teflon wrinkles

In Fig. S10 the same area is shown at two time points, showing the appearance of a nanobubble on the LIS. The nanobubble is flat (aspect ratio of width to height $w/h \approx 30$) and covers a significant portion of the scanned area. Point P in each map shows a force curve from the same spot before and after appearance of the nanobubble. Point Q shows a spot where there is no bubble and the lubricant layer remains unperturbed. The appearance of a region with a positive gradient is indicative of a nanobubble [6]. The algorithm is able to discern the different layers but does sometimes mislabel layers due to the small deflections involved. This results in the noise in the maps of the lubricant/air thickness.

Fig. S11 shows further examples of forces curves. Point 1 and Point 3 correspond to two regions where the AFM tip first encounters an air layer, evident by the increasing slope during the approach of the tip (starting at the blue line), and then is pulled down by the meniscus formed with the underlying lubricant layer (green line) before it makes hard contact with the solid substrate (red line). Point 2 is an area where the air layer displaced the lubricant because no pull-down of the tip is observed. Point 4 is a region where the solid substrate is exposed to the water, as neither repulsive nor attractive force is observed. Points 5 and 6 correspond to typical force curves of a lubricant infused surface as shown in [7].

Mapping of nanobubbles on superhydrophobic Teflon wrinkles

Fig. S12 shows a map and force curves of a (non-infused) superhydrophobic Teflon wrinkled surface under Milli-Q water ($c_{air} \sim 23.0 \pm 0.3$ mg kg^{-1}). The surface is in a partially collapsed Wenzel state, with only the regions of deeper topography in the top right of the map trapping air. As the cantilever deflections are small and the script sometimes mislabel features, noise seen and the occasional non-zero measured lubricant film thickness are visible, even though no lubricant is present on the surface.

Mapping of nanobubbles on infused wrinkles in water with high air content

Fig. S13 shows a map and force curves of a Teflon wrinkled surface infused with silicone oil and immersed in gassed water with $c_{air} \sim 44 \pm 4$ mg kg^{-1}. The large amount of air in the water results in a layer of air 100 nm to 300 nm thick covering almost completely the surface. Lubricant is seen in the valleys between the wrinkles.

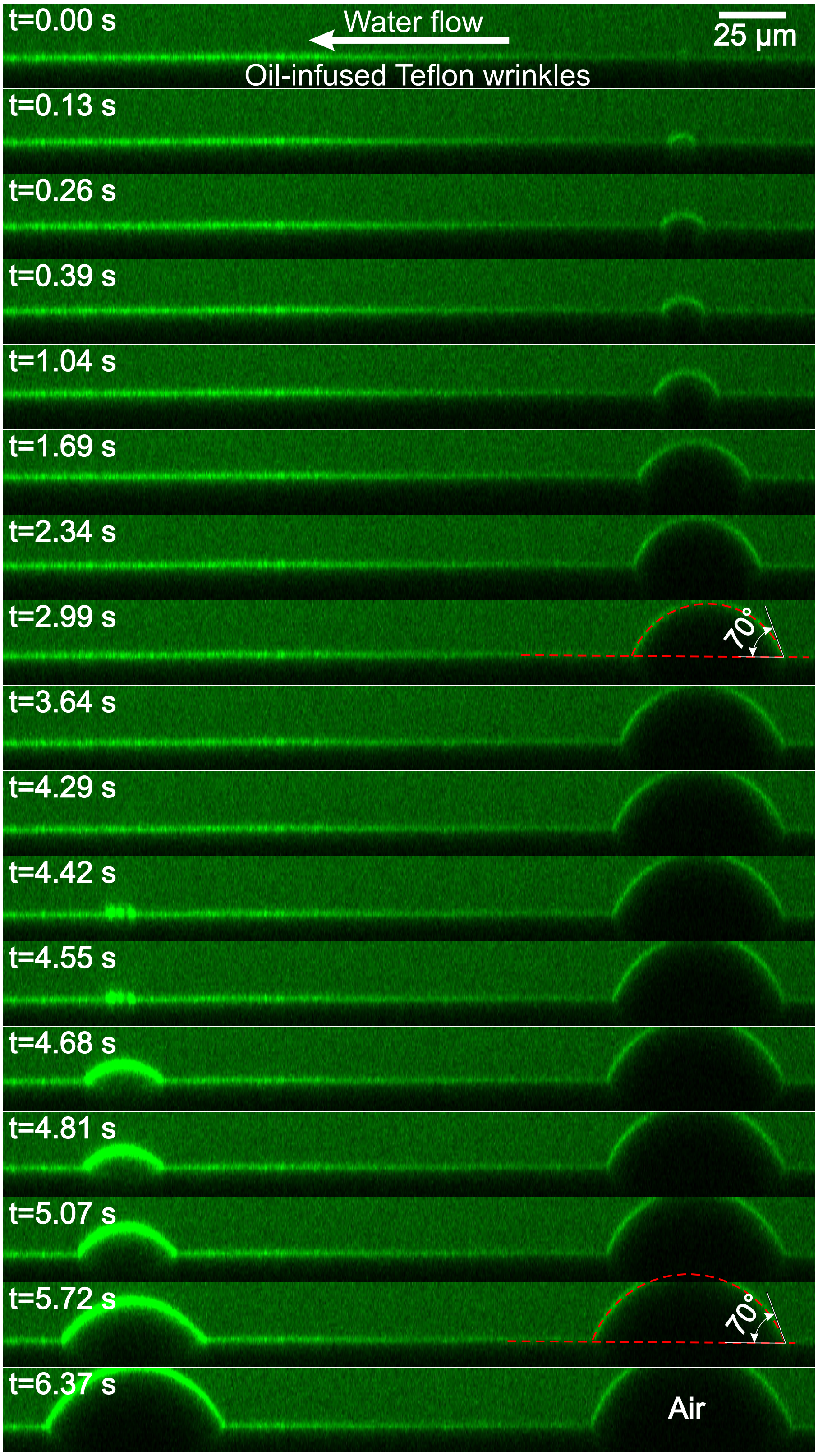

FIG. S9. Time sequence showing bubble nucleation on oil-infused Teflon wrinkles under highly gassed water ($c_{air} \sim 65 \pm 7$ mg kg^{-1}) at flow rate of 600 µL min^{-1}. Note that the time tag does not increase linearly.

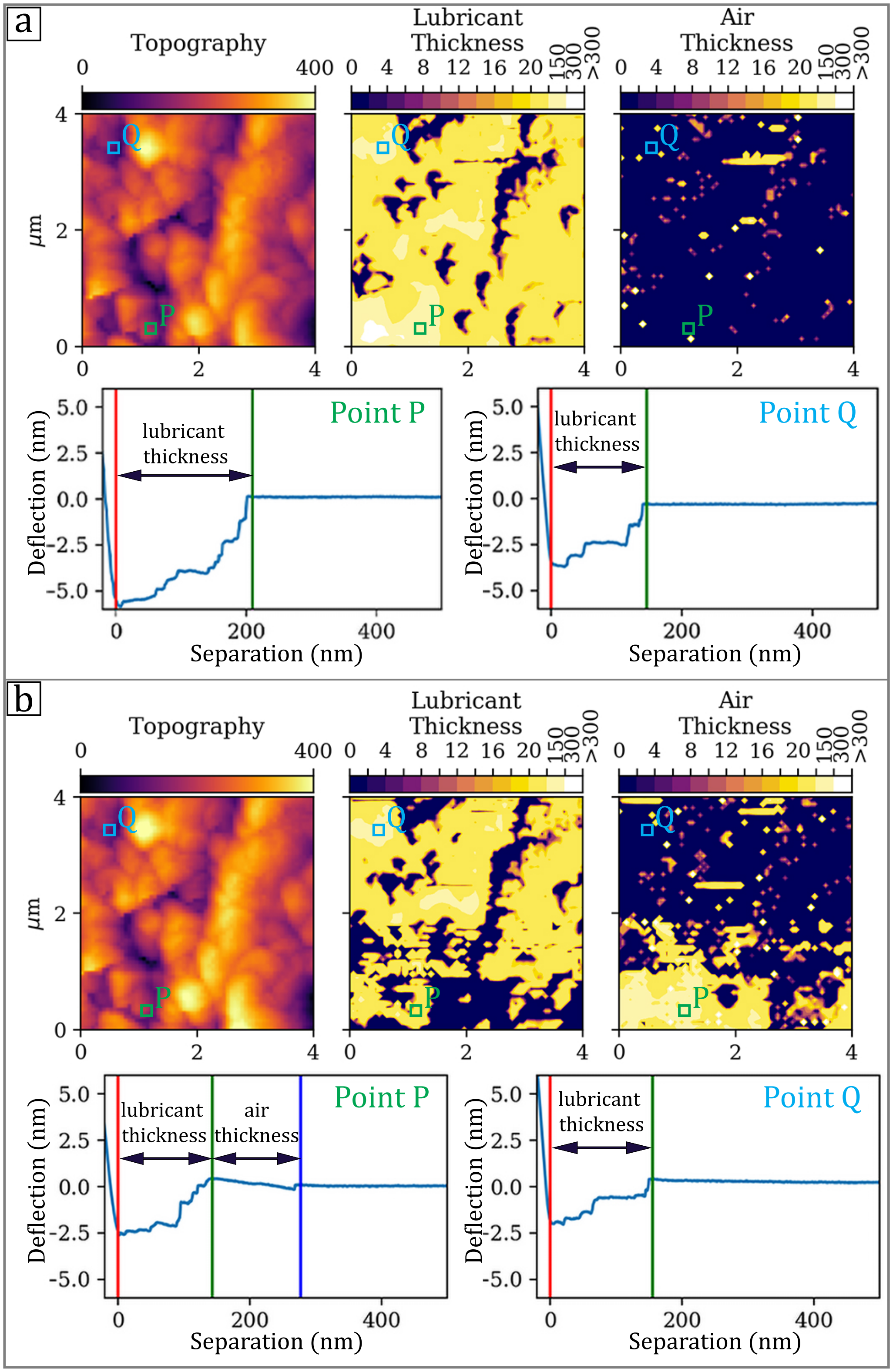

FIG. S10. AFM meniscus force map of infused Teflon wrinkles under Milli-Q water ($c_{air} \sim 23.0 \pm 0.3$ mg kg^{-1}), before (a) and after (b) the nucleation of a nanobubble in the lower left-hand corner. The left panel shows the wrinkle topography (red line in the force curves); the middle panel the thickness of the lubricant (green line); and the left panel the thickness of any air layer, as calculated by the analysis script. (b) was collected approx. 35 minutes after (a). The bubble is revealed by the positive deflection in the force curve (after the blue line), before the jump-in due to the lubricant layer. The color scale bars of the maps are all in nm.

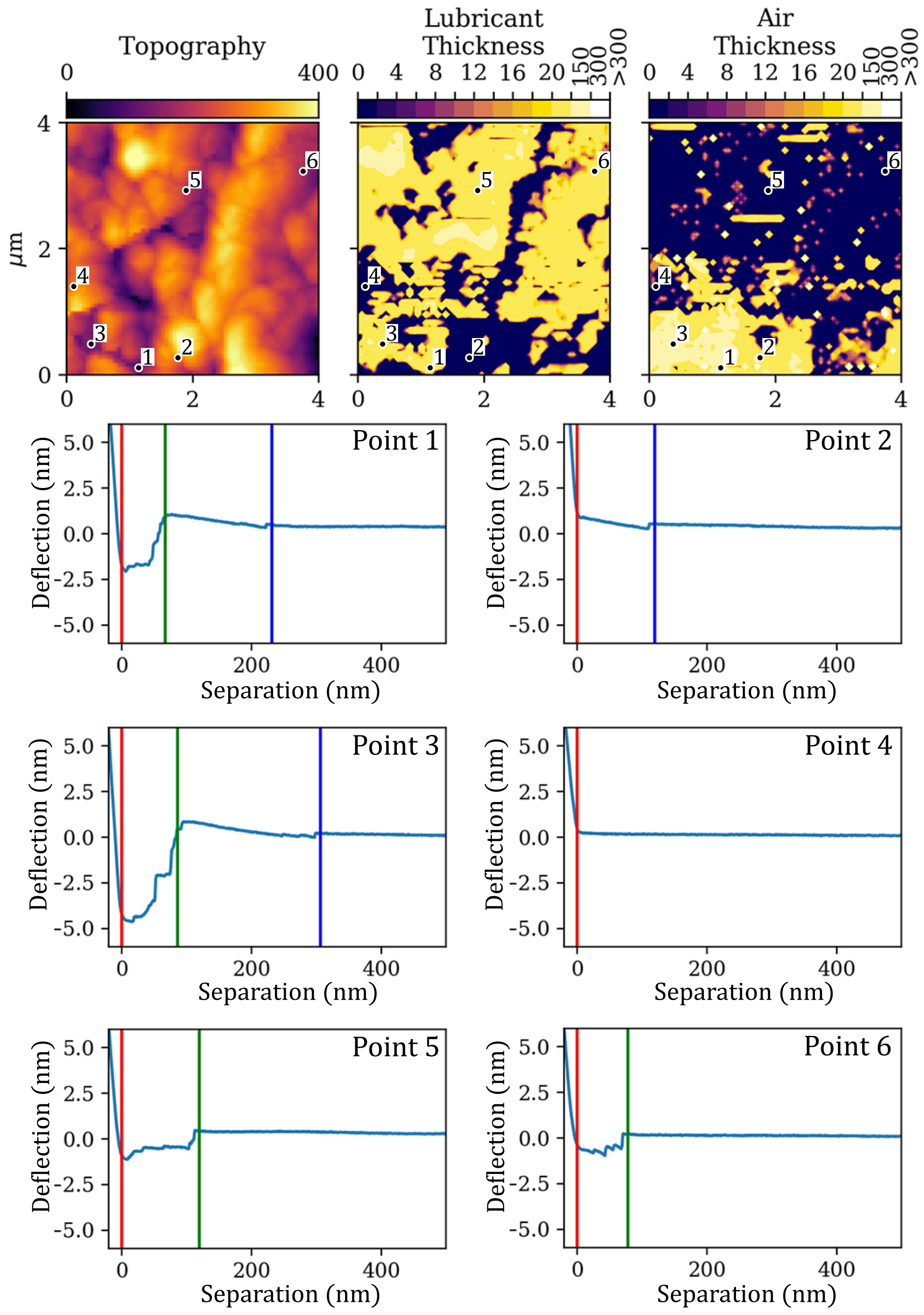

FIG. S11. Additional AFM meniscus force curves of infused Teflon wrinkles from the map shown in Figure S10(b)($c_{air} \sim 23.0 \pm 0.3$ mg kg^{-1}). The red line indicates the location of the substrate as detected by the analysis script; the green line indicates the location of the top of the lubricant layer as detected by the analysis script; the blue line indicates the location of the top of air layer as detected by the analysis script. The color scale bars of the maps are all in nm.

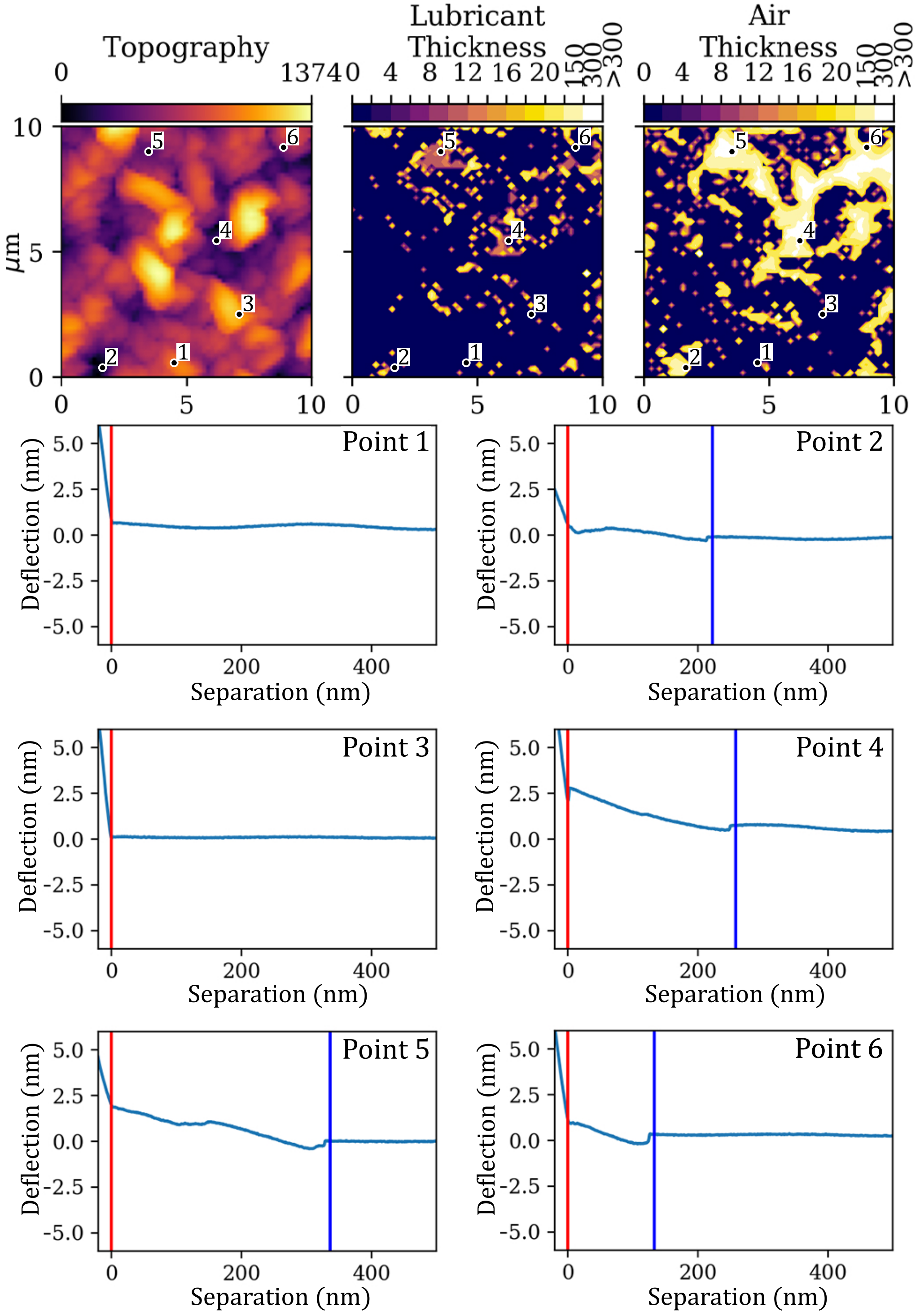

FIG. S12. Example AFM meniscus force map of a (non-infused) superhydrophobic Teflon wrinkled surface under Milli-Q water($c_{air} \sim 23.0 \pm 0.3$ mg kg^{-1}), with example force curves from the locations shown. The red line indicates the location of the substrate as detected by the analysis script; the blue line indicates the location of the top of air layer as detected by the analysis script. The color scale bars are all in nm.

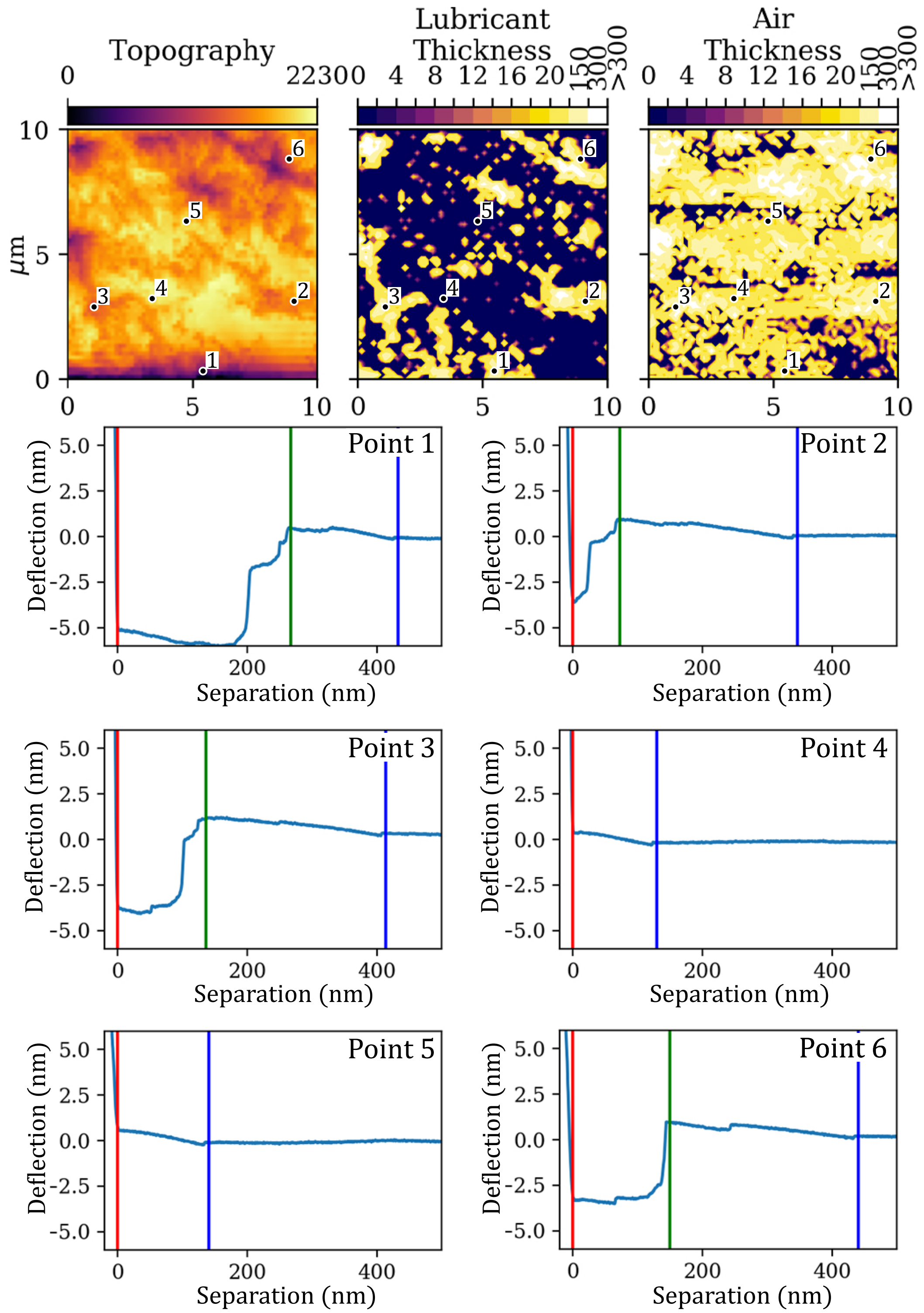

FIG. S13. Example maps and force curves of a Teflon wrinkled surface infused with silicone oil under gassed water ($c_{air} \sim 44{\pm}4$ mg kg^{-1}) with a large air layer. The red line indicates the location of the wrinkled substrate, the green line the top of the lubricant layer; the blue line the top of air layer as detected by the analysis script. The color scale bars of the maps are all in nm.

BUBBLE NUCLEATION AND THE THREE-LAYER APPARENT SLIP MODEL

In the apparent slip model, the working fluid flows over a lubricant film of uniform thickness h_o, in a bi-layer geometry as in Fig. S14a. Under this condition, the upper bound for the slip length is defined as $b_{max} = \frac{\mu_w}{\mu_o} h_o$. However, the nucleation of bubbles at the interface could lead to an increased slippage not captured by this model. A three-layer model including the presence of a gas layer between the working fluid and the lubricant is shown in Fig. S14b.

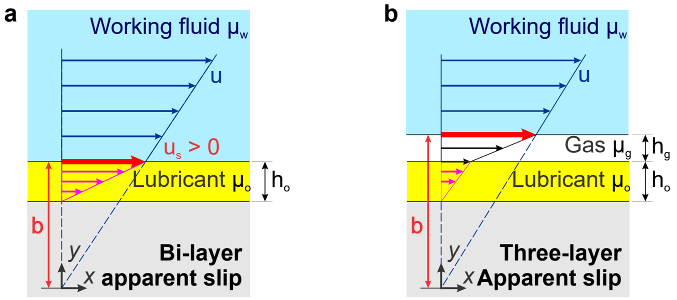

FIG. S14. Apparent slip model of a (a) bi-layer and (b) three-layer system.

For a Couette flow cell containing three layers of fluids of known viscosities and heights, as shown in Fig. S14b, and assuming that the velocities and shear stresses of the fluids at the interface match each other, the slip length can be computed as:

$$b = \frac{\mu_w(h_g\mu_o + h_o\mu_g)}{\mu_g\mu_o} \tag{4}$$

Here, h_o and h_g are the average height of the lubricant film and of the gas layer, respectively. The gas layer is assumed to be air ($\mu_g = 1.8 \times 10^{-5}$ Pa s). Assuming that the large slippage observed is due to the tri-layer model with a constant $h_o = 0.8$ µm, we calculated the gas layer average thickness required to fit the results. The results are shown in Table S7. In reality, we have demonstrated that isolated nanobubbles are present on the surface, with their distribution density increasing with increased air content in the flowing liquid.

TABLE S7. Estimation of gas film thickness h_g based on the experimental slip length b_{exp} (Fig. 3e, main text) . All these experiments were carried out at a constant $Ca = 0.001$ with an air content $c_{air} \sim 44 \pm 4$ mg kg^{-1}.

Viscosity ratio μ_w/μ_o	b_{exp} (µm)	h_g (nm)	Re	Shear rate (s^{-1})
0.1	5.2	103.5	13.194	3302
1.0	6.7	11.5	0.074	187
5.5	12.8	3.0	0.003	34
9.7	17.9	2.0	0.001	20

STUDY ON LUBRICANT RECIRCULATION WITHIN THE TEFLON WRINKLES

Normally, the recirculation of lubricant within the substrate topography explains the apparent slip observed in LIS. To show that this mechanism alone cannot explain our experimental results, a two-dimensional numerical model was solved using the two-phase flow interface of Comsol Multiphysics. The lubricant-liquid interface was tracked using the phase field method. The geometry consists of a rectangular domain (height $H = 5$ µm and length $L = 20$ µm). The bottom surface is a real Teflon wrinkled profile obtained from AFM measurements. The initial condition of the domain consists of a lubricant layer (thickness 1 µm, $\mu_o = 9.3$ mPa s) on top of the wrinkled profile, while the rest is filled with water ($\mu_w = 0.89$ mPa s). A Couette flow is induced by imposing a shear rate in the upper surface in the x direction, parallel to the horizontal axis of the geometry. The value of the shear rate ($\sim 2400\,\text{s}^{-1}$) was estimated to reflect the highest flow rate used in our experiments (800 µL min^{-1}). The left and right vertical walls of the domain

are defined as inlet and outlet, respectively. A no-slip boundary condition is assumed on all the liquid-solid interfaces and a wetted wall condition is applied to all the walls. The interfacial tension of the oil/water interface is $\gamma_{w/o} = 44$ mN m^{-1}. A transient simulation is carried out and set to run until it reaches the steady state in which no oil is depleted (this is confirmed by integrating the lubricant mass over time in the entire domain). Fig. S15 shows the results of the modelling, with recirculation zones observed in the pools between the Teflon wrinkles.

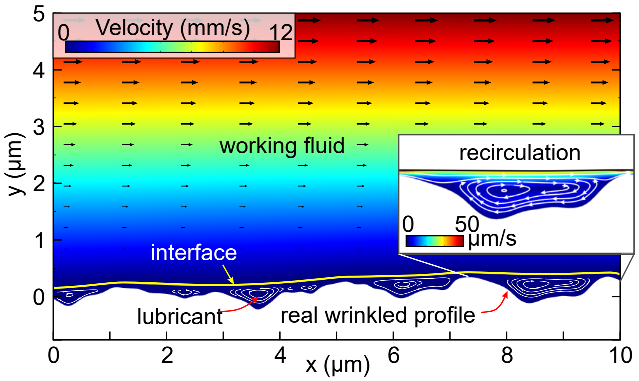

FIG. S15. Velocity field obtained from two-phase flow computational fluid dynamics simulations for a steady state Couette flow of water over Teflon wrinkles infused with 10 cSt silicone oil. The wrinkled substrate is taken from a real AFM map. The black arrows indicate the velocity direction and are proportional to the velocity magnitude. The deformation of the interface under the imposed shear is shown with the yellow line. The streamlines in the inset show the recirculation of lubricant.

[1] Gajasinghe, R. *et al.* Experimental study of PDMS bonding to various substrates for monolithic microfluidic applications. *J Micromech Microeng.* **24**, 075010 (2014).

[2] Butler, I. B., Schoonen, M. A. & Rickard, D. T. Removal of dissolved oxygen from water: A comparison of four common techniques. *Talanta* **41**, 211–215 (1994).

[3] Mortimer, C. H. The oxygen content of air-saturated fresh waters over ranges of temperature and atmospheric pressure of limnological interest. *SIL Communications, 1953-1996* **22**, 1–23 (1981).

[4] Scarratt, L. R., Zhu, L. & Neto, C. How slippery are SLIPS? Measuring effective slip on lubricated surfaces with colloidal probe atomic force microscopy. *Langmuir* **35**, 2976–2982 (2019).

[5] Scarratt, L. R., Hoatson, B. S., Wood, E. S., Hawkett, B. S. & Neto, C. Durable superhydrophobic surfaces via spontaneous wrinkling of Teflon AF. *ACS Appl. Mater. Interf.* **8**, 6743–6750 (2016).

[6] Walczyk, W. & Schönherr, H. Characterization of the interaction between AFM tips and surface nanobubbles. *Langmuir* **30**, 7112–7126 (2014).

[7] Peppou-Chapman, S. & Neto, C. Mapping depletion of lubricant films on antibiofouling wrinkled slippery surfaces. *ACS Appl. Mater. Interf.* **10**, 33669–33677 (2018).
